# Elk1 enhances inflammatory cell infiltration and exacerbates acute lung injury/acute respiratory distress syndrome by suppressing Fcgr2b transcription

**DOI:** 10.1186/s10020-024-00820-z

**Published:** 2024-04-22

**Authors:** Shiyou Wei, Dandan Ling, Jingui Zhong, Rui Chang, Xinyu Ling, Zhigang Chen, Ruowang Duan

**Affiliations:** 1 Department of Anesthesiology, Shanghai Pulmonary Hospital, School of Medicine, Tongji University, Shanghai, 200433 China; 2 Department of Anesthesiology, Fudan University Shanghai Cancer Center, Shanghai, 200032 China; 3 Department of General Surgery, Zhabei Central Hospital of Jing’an District, Shanghai, 200070 China; 4 Medical department, Shanghai Pulmonary Hospital, School of Medicine, Tongji University, Shanghai, 200433 China; 5 Department of Thoracic Surgery, Shanghai Pulmonary Hospital, School of Medicine, Tongji University, Shanghai, 200433 China; 6 Outcomes Research Consortium, Cleveland, OH USA

**Keywords:** Elk1, Fcgr2b, ARDS, ALI, Inflammatory cell infiltration

## Abstract

**Objective:**

Acute lung injury (ALI) and acute respiratory distress syndrome (ARDS) are associated with significant mortality rates. The role of Fcgr2b in the pathogenesis of ALI/ARDS is not fully elucidated. This study aimed to investigate the functions of Fcgr2b in ALI/ARDS and explore its underlying mechanisms.

**Methods:**

Methods: In this study, rat models of ARDS and pulmonary microvascular endothelial cell (PMVEC) injury models were established through the administration of lipopolysaccharide (LPS). The expression levels of Fcgr2b and Elk1 were quantified in both LPS-induced ARDS rats and PMVECs. Subsequent gain- and loss-of-function experiments were conducted, followed by comprehensive assessments of lung tissue for pathomorphological changes, edema, glycogen storage, fibrosis, and infiltration of inflammatory cells. Additionally, bronchoalveolar lavage fluid was analyzed for T-helper 17 (Th17) cell infiltration, inflammatory response, and microvascular permeability to evaluate lung injury severity in ARDS models. Furthermore, the activity, cytotoxicity, apoptosis, and angiogenic potential of PMVECs were assessed to gauge cell injury. The interaction between Elk1 and Fcgr2b was also examined to confirm their regulatory relationship.

**Results:**

In the context of LPS-induced ARDS and PMVEC injury, Fcgr2b expression was markedly reduced, whereas Elk1 expression was elevated. Overexpression of Fcgr2b led to a decrease in Th17 cell infiltration and mitigated lung tissue damage in ARDS models, in addition to reducing LPS-induced injury in PMVECs. Elk1 was found to suppress Fcgr2b transcription through the recruitment of histone 3 lysine 9 trimethylation (H3K9me3). Knockdown of Elk1 diminished Th17 cell infiltration and lung tissue damage in ARDS models, and alleviated LPS-induced injury in PMVECs, effects that were reversed upon Fcgr2b upregulation.

**Conclusion:**

Elk1 negatively regulates Fcgr2b transcription, thereby augmenting the inflammatory response and exacerbating lung injury in LPS-induced ALI/ARDS.

## Highlights

LPS-induced ARDS rats and PMVECs have low Fcgr2b level and high Elk1 level;

Fcgr2b overexpression mitigates LPS-induced ALI/ARDS in rats and PMVECs;

Elk1 knockdown mitigates LPS-induced ALI/ARDS in rats and PMVECs;

Elk1 represses Fcgr2b transcription by recruiting H3K9me3;

Elk1/Fcgr2b axis aggravates LPS-induced ALI/ARDS in rats and PMVECs.

## Introduction

ALI/ARDS is a biologically and clinically heterogeneous condition associated with multiple disease processes, leading to severe hypoxemia, decreased compliance, and increased non-hydrostatic extravascular lung water (Banavasi et al. 2021). The Berlin Definition classifies ARDS into three categories in accordance with the extent of hypoxemia: mild (arterial partial pressure of oxygen [PaO2]/fraction of inspired oxygen [FiO2] between 200 and 300 mm Hg), moderate (PaO2/FiO2 between 100 and 200 mm Hg), and severe (PaO2/FiO2 ≤ 100 mm Hg) (Ranieri et al. 2012). Patients diagnosed with ALI/ARDS exhibit a high mortality rate, with observed in-hospital death rates amounting to 26.5% for mild cases, 31.8% for moderate cases, and 60.0% for severe cases. Furthermore, the three-year mortality rates escalate to 43.5% for mild ARDS, 46.9% for moderate ARDS, and 71.1% for severe ARDS, respectively (Parhar et al. 2019). The predominant approach to managing ALI/ARDS involves supportive care, including mechanical ventilation and fluid management. However, the prognosis for the majority of ALI/ARDS patients remains poor due to the limited efficacy of current treatment modalities (Liu et al. 2022a, b). Therefore, investigating the molecular mechanisms underlying ALI/ARDS is crucial for the development of therapeutic strategies aimed at the effective management of this condition.

The Fragment Crystallizable Gamma Receptor IIB (Fcgr2b) constitutes a member of the Fc receptor (FcR) family, a group of proteins expressed on immune cells. These receptors engage with the Fc region of Immunoglobulin G (IgG), playing a pivotal role in modulating the interplay between the adaptive and innate immune responses (Sun et al. 2023). Impairment of Fcgr2b expression or function is related to lupus in human and mice, particularly by affecting plasma cell differentiation and autoantibody production (Barlev et al. 2022). According to reports, Fcgrs and virus-specific IgG facilitate the infection of a subset of monocytes by severe acute respiratory syndrome coronavirus 2 in patients (Junqueira et al. 2022). However, no studies have yet addressed the action of Fcgr2b in ALI/ARDS, which requires examination. In Divyesh Patel et al. reserach, SYK inhibition also restored the Th17/Treg balance via decreased Th17 and increased Treg responses, as evidenced by decreased IL-17 and ror-γ levels, therefore ameliorates fungal induced airway inflammation (Patel et al. 2018). Furthermore, the erythroblast transformation specific (ETS) domain-containing protein-1 (Elk1), a transcription factor potentially regulating Fcgr2b, was identified through chromatin immunoprecipitation sequencing (ChIP-seq) analysis. Elk1, a member of both the ternary complex factor subfamily and the ETS family, plays essential roles in the regulation of cellular survival, differentiation, growth, and various other biological processes (Yu et al. 2021). The most popular studies of Elk1 are exploring its roles in cancers, like glioma, bladder, colorectal, and cervical cancers (Wang et al. 2020; Ma et al. 2021; Shen et al. 2020; Huang and Luo 2021). Leikauf et al. identified Elk1 and other candidate genes related to enhanced susceptibility to ALI in mice using a genetic/genomic approach (Leikauf et al. 2011) .Significantly, the upregulation of G-protein-coupled receptor 43 has been shown to diminish the inflammatory response and apoptosis, thereby mitigating ALI in lipopolysaccharide (LPS)-induced mice through the modulation of the c-Jun N-terminal kinase/Elk1 pathway (Xu et al. 2022), indicating that Elk1 performs a role in ALI/ARDS. Collectively, the present research was committed to discussing the actions of Fcgr2b on ALI/ARDS and its mechanism, so as to offer novel insights for the management of ALI/ARDS.

## Materials and methods

### Establishment of ARDS rat model

Male Wistar rats, sourced from Vital River Laboratories, Beijing, China, underwent an acclimatization period of one week before the commencement of experiments and were systematically divided into seven groups: Control, ARDS, adeno-associated virus (AAV) negative control (NC), AAV-Fcgr2b, AAV-short hairpin RNA (sh)-NC, AAV-sh-Elk1, and AAV-sh-Elk1 + AAV-sh-Fcgr2b. To induce anesthesia, the rats received an intraperitoneal injection of 10% chloral hydrate (250 mg/kg). For the establishment of the ARDS model, 10 mg/kg of LPS was administered intratracheally to the Wistar rats, whereas an equivalent volume of saline was injected as a control measure. The adeno-associated virus interventions were executed via tail vein injection one week prior to LPS administration. Twelve hours post-LPS induction, the rats were anesthetized again with an intraperitoneal injection of 10% chloral hydrate (250 mg/kg). Arterial blood samples were then extracted from the carotid artery for subsequent analysis with a blood gas analyzer and for the determination of PaO2 to calculate the PaO2/FiO2 ratio. After the arterial blood collection, euthanasia was carried out using an overdose of sodium pentobarbital (Lai et al. 2016). All experiments involving animals were ratified by the Ethics Committee for Animal Experiments of Shanghai pulmonary hospital.

### Collection of bronchoalveolar lavage fluid (BALF)

Subsequent to LPS administration, the Wistar rats received an intratracheal injection of 2 mL phosphate-buffered saline (PBS) combined with ethylene diamine tetraacetic acid for the purpose of bronchoalveolar lavage fluid (BALF) collection. The percentage of Th17 cells within the BALF was ascertained through flow cytometry. Protein levels in the BALF were quantified utilizing bicinchoninic acid (BCA) protein assay kits (P0010, Beyotime, Shanghai, China). Furthermore, the total cell count in the BALF was determined via the use of hemocytometer counting chambers.

### Lung wet-to-dry weight (W/D) weight ratio

Lung W/D weight ratio was applied to evaluate the pulmonary edema. After euthanasia of rats, the lung tissues were obtained, cleaned using absorbent paper to remove exudate and residual blood from the surface, and weighed (wet weight). Next, the lung tissues were subjected to 48-h drying in a 65 °C oven and weighed (dry weight). Finally, the W/D weight ratio was calculated.

### Vascular permeability

To assess pulmonary vascular permeability, the accumulation of Evans blue dye within the tissue was quantified. Evans blue (25 mg/kg, obtained from Sigma-Aldrich) was administered intravenously via the tail vein 2 h prior to lung collection. The extraction of Evans blue from the lungs was facilitated by incubating the tissue in 1 ml of formamide at 60 °C for 18 h. This was followed by perfusion with 5 ml of phosphate-buffered saline (PBS), homogenization in 1 ml PBS, and subsequent dual washes. The supernatant was then isolated through centrifugation at 5000 g for 30 min.

### Immunohistochemistry

Following an overnight fixation in 4% paraformaldehyde at 4 °C, lung tissues underwent a series of preparatory steps including dehydration in graded alcohols, clarification in xylene, embedding in paraffin, and sectioning. Subsequently, the sections were deparaffinized in xylene, rehydrated through a graded alcohol series, and submerged in 3% H2O2 for 8 min to inhibit endogenous peroxidase activity. Antigen retrieval was then performed by subjecting the sections to microwave boiling in a citric acid-based solution three times. After cooling to ambient temperature, the sections were incubated with primary antibodies overnight at 4 °C within a humidified chamber, followed by incubation with a horseradish peroxidase (HRP)-conjugated secondary antibody (1:1000, ab205719/ab6721, Abcam, Cambridge, UK) at room temperature for 30 min. Development of the sections was achieved using a diaminobenzidine substrate, with the reaction halted by rinsing in tap water. The sections were counterstained with hematoxylin, subjected to a dehydration and clearing process, and finally mounted with neutral balsam. Microscopic examination facilitated the assessment of protein expression, quantified by the product of the H score (representing the percentage of area expressing the protein, ranging from 0 to 100%) and the intensity score (categorized into 4 levels: 0 = no staining, 1 = mild staining, 2 = moderate staining, and 3 = strong staining). The primary antibodies used were directed against Fcgr2b (1:100, 550,270, BD Biosciences, Bedford, MA, USA), retinoid-related orphan receptor-gammat (RORγt, 1:100, orb500718, Biorbyt, Cambridge, UK), and Elk1 (1:100, sc-365,876, Santa Cruz Biotechnology, Santa Cruz, CA, USA). Two pathologists, blinded to the study, used a multiheaded microscope to assess protein expression. They evaluated the intensity and distribution to agree on the H-score, calculated as ΣPi (i + 1), where i indicates the intensity of stained cells (0: negative, 1: weak positive, 2: moderate positive, 3: strong positive), and Pi represents the percentage of stained cells (Hsiao et al. 2023).

### Immunofluorescence analysis

Pulmonary tissue sections underwent permeabilization using 0.15% Triton X-100 for a duration of 15 min, followed by blocking in 5% normal goat serum dissolved in PBS for one hour. They were then subjected to overnight incubation at 4 °C with antibodies targeting Fcgr2b or cleaved-Caspase-3. This step was succeeded by the application of secondary antibodies for a period of 30 min at ambient temperature, prior to the nuclei being stained with DAPI (Fluka) for 10 min. Immunofluorescence imaging was accomplished utilizing an LSM 880 confocal microscope (Zeiss).

### Periodic acid-Schiff (PAS) staining

Rat lung tissue sections were initially processed through a dewaxing phase, followed by an oxidation step involving treatment with 1% periodic acid for 10 min. Subsequent to oxidation, the sections underwent thorough washing with distilled water. For the staining process, Schiff’s Reagent (Sigma-Aldrich, Merck KGaA, Darmstadt, Germany) was utilized for 20 min, succeeded by counterstaining with hematoxylin for 10 min. These procedures were conducted with precision at a strictly maintained temperature of 20 °C. Post-staining, the sections were subjected to examination and documentation using an optical microscope, facilitating detailed analysis.

### Masson’s trichrome staining

Rat lung tissue sections underwent dewaxing in xylene, followed by rehydration through a graded ethanol series. Subsequently, the sections were stained with Weigert’s iron hematoxylin and differentiated in 1% hydrochloric acid in ethanol. They were then rinsed under running water to restore the blue hue, stained with ponceau-acid fuchsin, and treated with a 1% phosphomolybdic acid solution for 3 min. Following this, the sections were counterstained with aniline blue for 5 min and differentiated in glacial acetic acid for 1 min. After these staining procedures, the sections were dehydrated in 95% followed by absolute ethanol for 5 min each, cleared in xylene, and mounted with neutral balsam for microscopic examination to evaluate tissue fibrosis.

### Flow cytometry

Cells in BALF were washed with PBS and resuspended in buffer. Then, cells underwent 30-min incubation with CD4-fluorescein isothiocyanate (11-0041-82, Invitrogen, Tokyo, Japan) and Interleukin (IL)-17 A-PE (12-7177-81, Invitrogen) at 10 °C, washing, resuspension in buffer, and detection on a flow cytometer.

### Hematoxylin-eosin (HE) staining

Paraffin-embedded lung tissue sections were processed through deparaffinization, rehydration, and subsequently stained with hematoxylin for 5 min. Following staining, the sections were rinsed with tap water and differentiated in 1% hydrochloric acid in ethanol for 3 s. Once the sections had reverted to a blue hue upon exposure to tap water, they were stained with eosin for 3 min. The final steps involved clearing the sections in xylene, mounting with neutral gum, and subsequent imaging and examination under a microscope.

### Enzyme-linked immunosorbent assay (ELISA)

BALF was centrifuged at 500 × g for 5 min to harvest the supernatant. IL-17 and IL-6 levels in BALF were determined via IL-17 (ml037365, Shanghai Enzyme-linked Biotechnology, Shanghai, China) and IL-6 (ab234570, Abcam) kits.

### Cell cultivation and processing

Rat pulmonary microvascular endothelial cells (PMVECs, Procell, Wuhan, China) were cultured in complete medium specifically designed for rat PMVECs (CM-R001, Procell) within a humidified incubator set at 5% CO2 and 37 °C. Following enzymatic digestion, PMVECs were harvested and plated into 6-well plates at a density of 1 × 10^5 cells per well. Subsequently, PMVECs were transduced with various lentiviral constructs (VectorBuilder, Guangzhou, China) for a duration of 48 h. To establish an in vitro model of ARDS, PMVECs were exposed to 1 µg/mL LPS for 4 h to induce cellular injury.

### Reverse transcription-quantitative polymerase chain reaction (RT-qPCR)

Total RNA was extracted from rat PMVECs and lung tissues utilizing Trizol reagent (Invitrogen) and its concentration was determined using the Hifair® III One Step RT-qPCR SYBR Green Kit (11143ES70, Yeasen Biotechnology, Shanghai, China). Relative gene expression levels were quantified employing the 2^−ΔΔCt^ method, with glyceraldehyde-3-phosphate dehydrogenase (GAPDH) serving as the internal control for normalization. The specific primers used in this study are listed as follows: for Fcgr2b, the forward primer is TTCCGAAGGCTGTGGTGAAA and the reverse primer is TCCCTTCGCACATCAGTGTC; for Elk1, the forward primer is AGGAAGCTGAGGCAAGAGTTC and the reverse primer is CGCTCACCTTGCGGATGATA; for GAPDH, the forward primer is GCATCTTCTTGTGCAGTGCC and the reverse primer is GATGGTGATGGGGTTTCCCGT.

### Cell counting kit (CCK)-8 assay

Cell viability was assessed using the CCK-8 assay (ab228554, Abcam). Briefly, following treatment with LPS, PMVECs were incubated with 10 µL of CCK-8 solution at 37 °C in the dark for 2 h. Subsequently, the absorbance was measured at 460 nm using a microplate reader.

### Measurement of lactate dehydrogenase (LDH)

The LDH kit (C0016, Beyotime) was applied to evaluate the LPS-induced cytotoxicity. Following LPS treatment, PMVECs received 5-min centrifugation at 500 × g. The supernatant was added with the LDH assay reagent for LDH measurement. The cytotoxicity of PMVECs was calculated per the formula: cytotoxicity (%) = (absorbance of treated samples - absorbance of control)/(absorbance of maximum enzyme activity of cells - absorbance of control) × 100%.

### TdT-mediated dUTP-biotin nick end-labeling (TUNEL)

The TUNEL assay (40307ES50, Yeasen Biotechnology) was employed to evaluate LPS-induced apoptosis in PMVECs. Initially, following enzymatic digestion, PMVECs were collected for slide preparation. Post-LPS treatment, the cells were fixed with 4% paraformaldehyde at room temperature for 30 min and permeabilized with 0.3% Triton X-100 for 5 min at room temperature. Subsequently, PMVECs were incubated with 100 µL of Equilibration Buffer for 30 min at room temperature, followed by incubation with TdT incubation buffer for 1 h at 37 °C in a humidified chamber away from light. After washing with PBS, the nuclei were stained with 4’,6-Diamidino-2-Phenylindole (DAPI) for 5 min. The enumeration of TUNEL-positive cells was conducted using a fluorescence microscope, and the proportion of apoptotic cells was quantitatively analyzed.

### Tubule formation assay

LPS-treated rat PMVECs at a density of 1 × 10^4 cells per well were plated onto 96-well plates pre-coated with Matrigel and then incubated for 12 h in a humidified atmosphere containing 5% CO2 at 37 °C. Subsequent to incubation, tubule formation was visualized and captured using microscopy. The total length of the tubules was quantified utilizing Image J software along with the angiogenesis analyzer plug-in. The comparative analysis of tubule length between the two groups facilitated the evaluation of the angiogenic capacity of PMVECs.

### Western blot

Total proteins from PMVECs and lung tissues were isolated using radio-immunoprecipitation assay lysis (20–188, Merck, Zurich, Switzerland), quantified using BCA, boiled in sample preparing buffer to prepare samples, and separated using sodium dodecyl sulfate polyacrylamide gel electrophoresis. The separated proteins were transferred to PVDF membranes, which were sealed in 3% skimmed milk for 30 min and probed with diluted primary antibodies overnight at 4 °C. On the next day, PVDF membranes received washing and 1-h probing with HRP-coupled secondary antibody (1:5000, ab205719, Abcam) at room temperature. The used antibodies are as follows: Fcgr2b (1:1000, 550,270, BD Biosciences), vascular endothelial (VE)-cadherin (1:3000, sc-9989, Santa Cruz Biotechnology), β-catenin (1:3000, 13-8400, Invitrogen), Elk1 (1:1000, sc-365,876, Santa Cruz Biotechnology), GAPDH (1:5000, ab8245, Abcam).

### ChIP-qPCR

ChIP kit (ab185913, Abcam) was employed for ChIP assay. PMVECs were cross-linked with formaldehyde for 15 min before 10-min glycine treatment for formaldehyde bursting. Following lysis with lysis buffer, PMVECs were sonicated to cut the chromatin into 200–1000 base pairs. Then, the mixture was immunoreacted with Elk1 (1:100, sc-365,876, Santa Cruz Biotechnology), histone 3 lysine 9 trimethylation (H3K9me3, 1:50, NBP1-30141, Novus Biologicals, Centennial, CO, USA) or IgG. After DNA and protein mixtures received elution with DNA release buffer and de-crosslinking with proteinase K, DNA was purified per the kit, and the enrichment of Fcgr2b promoter was measured by qPCR.

### Dual-luciferase reporter assay

Fcgr2b promoters were inserted into the pGL3-basic vectors to construct the promoter luciferase reporter vectors, which were co-transfected with sh-NC/sh-Elk1 into PMVECs using FuGENE® 6 Transfection Reagent (E2691, Promega, Madison, WI, USA). Forty-eight h after transfection, the transcriptional regulation of Elk1 to Fcgr2b was assessed via Dual-Glo® Luciferase Assay System (E2920, Promega) by measuring cellular luciferase activity.

### Statistical analysis

Data were analyzed using GraphPad Prism 8.0 statistical software, and values were denoted as mean ± standard deviation. Differences between two groups were compared using the *t*-test, and differences among multiple groups were compared using one-way or two-way analysis of variance with Tukey’s post-hoc test. Differences were statistically significant when *P* < 0.05.

## Results

### Fcgr2b expression was low in ARDS rats

**Fig. 1:**
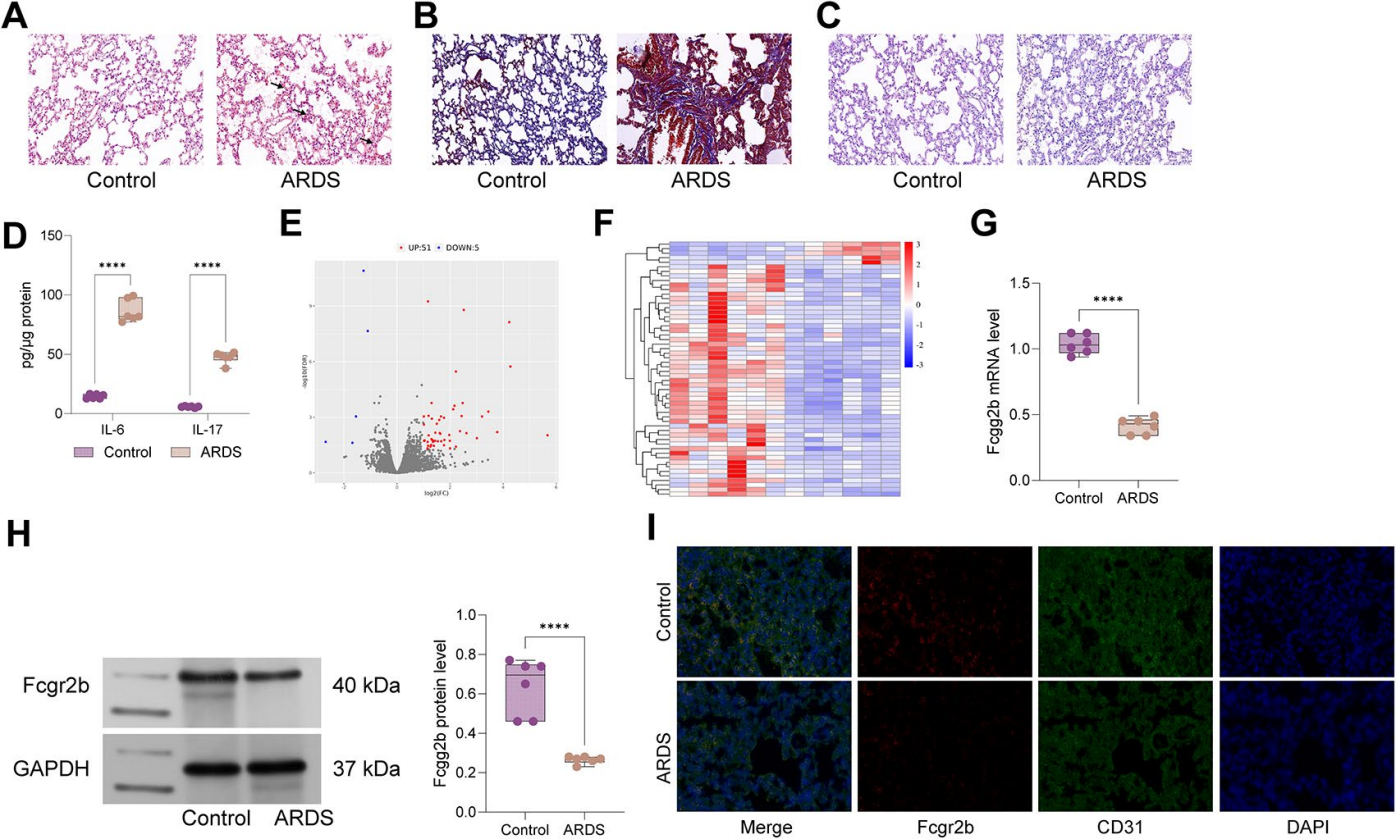
Fcgr2b is lowly expressed in ARDS rats. *Notes* **A**, HE staining to measure histological alterations in rat lung tissues; **B**, PAS staining to examine glycogen accumulation in rat lung tissues; **C**, Masson’s staining to evaluate the level of fibrous deposition in rat lung tissues; **D**, ELISA to detect the levels of inflammatory factors IL-17 and IL-6 in rat BALF; **E**-**F**, RNA-seq analysis of heat map and volcano map of differentially expressed genes in rat lung tissues; **G**-**H**, RT-qPCR and western blot to test Fcgr2b mRNA and protein expression in rat lung tissues. **I**, dual-labeled immunofluorescence to assess the fluorescence intensity of Fcgr2b and CD31 in lung tissue. Differences between two groups were compared using the *t*-test (**A**-**B**), and differences among multiple groups were compared using one-way or two-way analysis of variance with Tukey’s post-hoc test. ARDS, acute respiratory distress syndrome; BALF, bronchoalveolar lavage fluid

LPS was administered to rats to establish an ARDS model. Histological analysis revealed significant immune cell infiltration, increased glycogen accumulation, and enhanced fibrotic changes in the pulmonary tissues of these rats (Fig. 1A-C). Furthermore, the levels of inflammatory cytokines in the bronchoalveolar lavage fluid (BALF) of these rats were evaluated, demonstrating a pronounced increase in IL-17 and IL-6 concentrations (Fig. 1D), indicating the successful establishment of the ARDS model via LPS administration. RNA-seq analysis was conducted to identify differentially expressed mRNAs in the ARDS model, revealing a notable reduction in Fcgr2b expression in the lungs of ARDS-affected rats (Fig. 1E-F). This observation was corroborated by Western blot and RT-qPCR analyses, which also indicated a significant decrease in Fcgr2b expression in the pulmonary tissues of ARDS rats (Fig. 1G-H). Subsequently, we employed dual-labeled immunofluorescence to assess the fluorescence intensity of Fcgr2b in rat lung tissue. We observed a significant reduction in the fluorescence intensity of Fcgr2b in ARDS (Fig. 1I).

### Overexpression of Fcgr2b reduced Th17 cell infiltration in ARDS rats

**Fig. 2:**
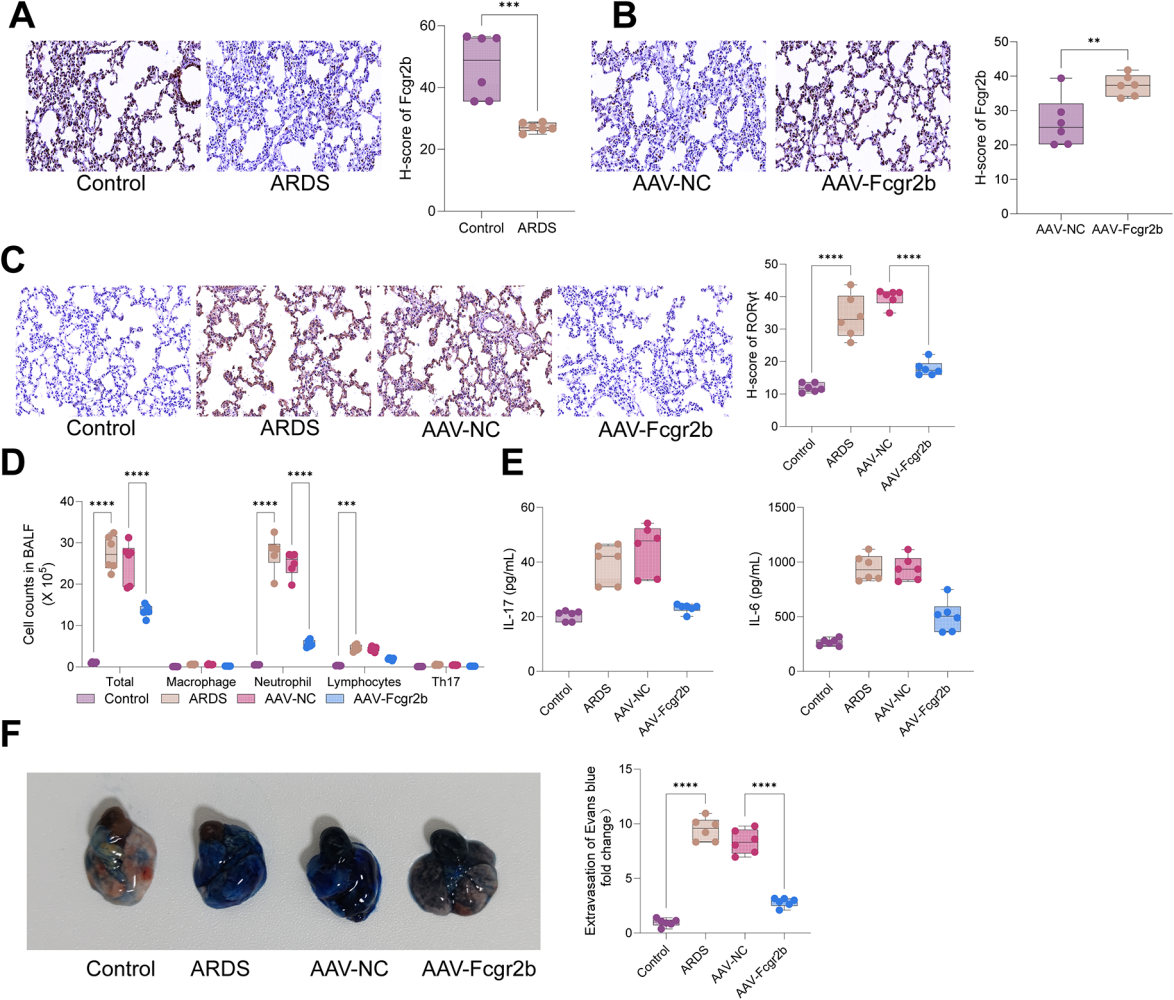
Th17 cell infiltration in ARDS rats is reduced by overexpressing Fcgr2b. *Notes* **A**, Immunohistochemistry to examine Fcgr2b expression in lung tissues of ARDS rats; **B**, immunohistochemistry to examine Fcgr2b expression in lung tissues of AAV-Fcgr2b-treated ARDS rats; **C**, immunohistochemistry to measure RORγt expression in lung tissues of each group of rats; **D**, Flow cytometry to determine the cell counts of Macrophage, Neutrophils, Lymphocytes and Th17 cells in BALF of each group of rats; **E**, ELISA to detect IL-17 and IL-6 expression in BALF of each group of rats. **F**, Photographs showing Evans blue accumulation in lung tissues (*n* = 6/group). Differences between two groups were compared using the *t*-test (**A**-**B**), and differences among multiple groups were compared using one-way or two-way analysis of variance with Tukey’s post-hoc test (**C**-**E**). AAV, adeno-associated virus; ARDS, acute respiratory distress syndrome; BALF, bronchoalveolar lavage fluid

Following LPS-induced ARDS, immunohistochemical analysis revealed a decrease in Fcgr2b expression in the lung tissues of ARDS-afflicted rats (Fig. 2A). To investigate the effect of Fcgr2b on ARDS, rats were administered adeno-associated virus (AAV) vectors encoding Fcgr2b prior to LPS induction. The findings demonstrated that AAV-mediated Fcgr2b overexpression significantly increased Fcgr2b levels in the lung tissues of ARDS rats (Fig. 2B). Immunohistochemical assessments showed that the expression of RORγt was markedly elevated in the lung tissues of ARDS rats; however, AAV-Fcgr2b substantially attenuated RORγt expression in these tissues (Fig. 2C). Furthermore, flow cytometric analysis indicated an increase in the proportion of Th17 cells in the BALF of ARDS rats, which was notably reduced following AAV-Fcgr2b treatment (Fig. 2D). ELISA results revealed that AAV-Fcgr2b significantly diminished the levels of IL-17 and IL-6 in the BALF of ARDS rats (Fig. 2E). In summary, overexpression of Fcgr2b decreased Th17 cell infiltration in ARDS rats. Additionally, Evans blue dye assay outcomes highlighted a significant decrease in lung tissue permeability in ARDS rats upon Fcgr2b overexpression (Fig. 2F).

### Overexpression of Fcgr2b ameliorated lung tissue injury in ARDS rats

**Fig. 3:**
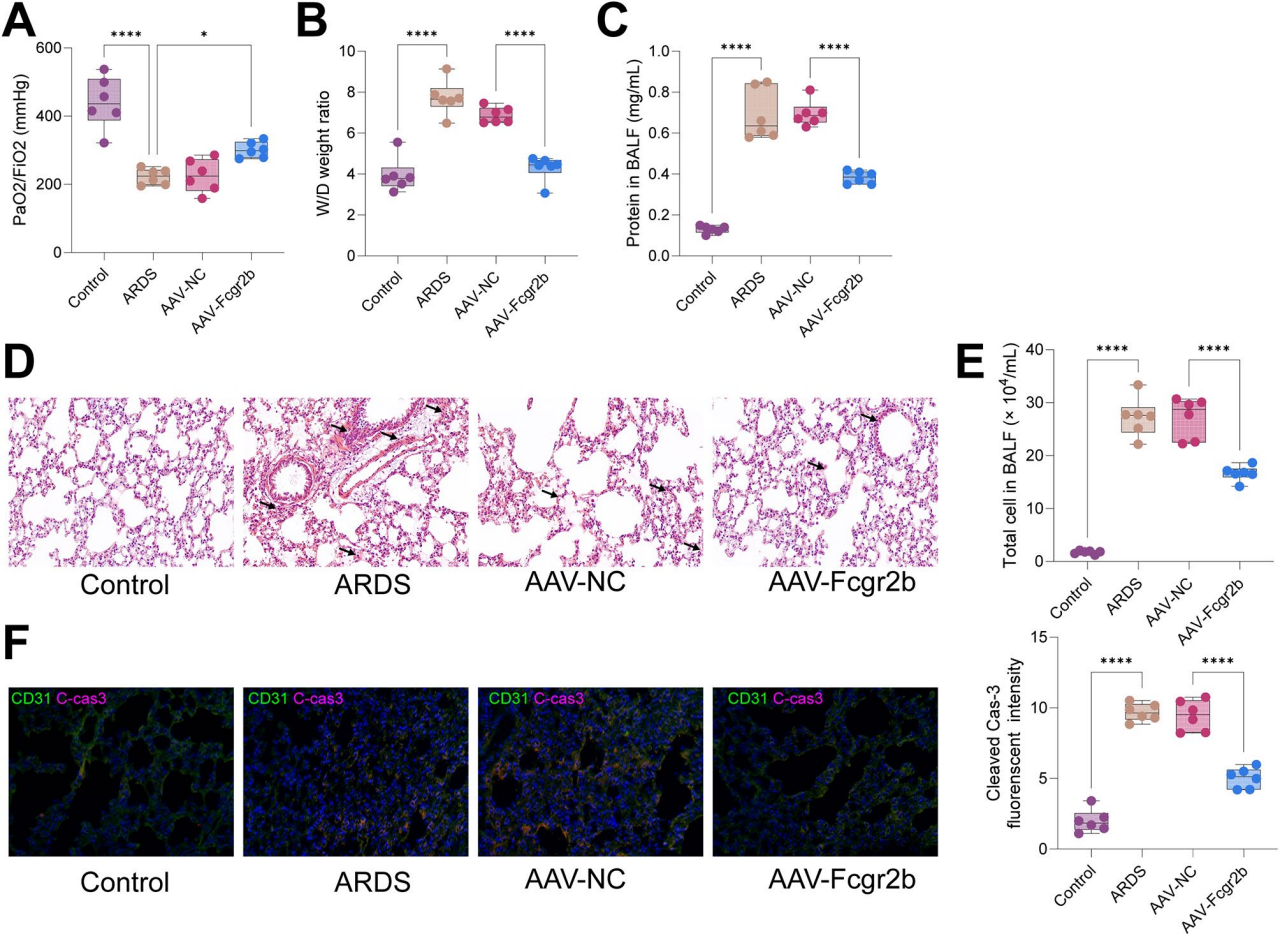
Lung tissue injury in ARDS rats is alleviated by overexpressing Fcgr2b. *Notes* **A**, The PaO2/FiO2 ratio of rats in each group; **B**, the lung W/D weight ratio of rats in each group; **C**, measurements of protein concentration in the BALF of rats in each group; **D**, HE staining to examine the pathological morphology of rat lung tissues in each group; **E**, examinations of the total number of cells in the BALF of rats in each group. **F**, dual-labeled immunofluorescence to assess the fluorescence intensity of Cleaved Cas-3 and CD31 in lung tissue. Differences among multiple groups were compared using one-way or two-way analysis of variance with Tukey’s post-hoc test (**A**-**C**, **E**). ARDS, acute respiratory distress syndrome; PaO2/FiO2, arterial partial pressure of oxygen/fraction of inspired oxygen; W/D, wet/dry; BALF, bronchoalveolar lavage fluid

As delineated in Fig. 3A, a significant reduction in the PaO2/FiO2 ratio was observed in ARDS-afflicted rats compared to their control counterparts, while administration of AAV-Fcgr2b markedly ameliorated the PaO2/FiO2 ratio in the ARDS group. In comparison to control animals, ARDS rats demonstrated a pronounced elevation in the lung wet/dry (W/D) weight ratio, indicative of exacerbated pulmonary edema. Treatment with AAV-Fcgr2b mitigated the severity of lung tissue edema in the ARDS cohort (Fig. 3B). The integrity of the pulmonary microvascular system was evaluated by quantifying protein levels in the BALF, revealing increased protein concentrations and compromised microvasculature in ARDS rats, whereas AAV-Fcgr2b therapy resulted in decreased BALF protein levels, suggesting restored microvascular permeability in treated ARDS rats (Fig. 3C). HE staining illustrated that lung tissues from control rats exhibited a well-preserved architecture with no significant pathologic alterations. Conversely, lung specimens from ARDS rats displayed thickened alveolar septa and extensive infiltration by inflammatory cells. Notably, AAV-Fcgr2b treatment significantly reduced the extent of tissue injury and inflammatory cell infiltration in the lungs of ARDS rats (Fig. 3D). To assess the inflammatory milieu within the ARDS model, total cell counts in BALF were performed, showing a substantial increase in cellularity in ARDS rats, which was significantly reduced following AAV-Fcgr2b intervention (Fig. 3E).Subsequently, we utilized immunofluorescence to detect the fluorescence intensity of the apoptosis marker Cleaved-Cas-3 in lung tissue. We observed a significant alleviation of apoptosis in rat lung tissue following overexpression of Fcgr2b (Fig. 3F). Overall, Fcgr2b overexpression improved lung tissue injury in ARDS rats.

### Overexpression of Fcgr2b depressed LPS-induced cell injury in PMVECs

**Fig. 4:**
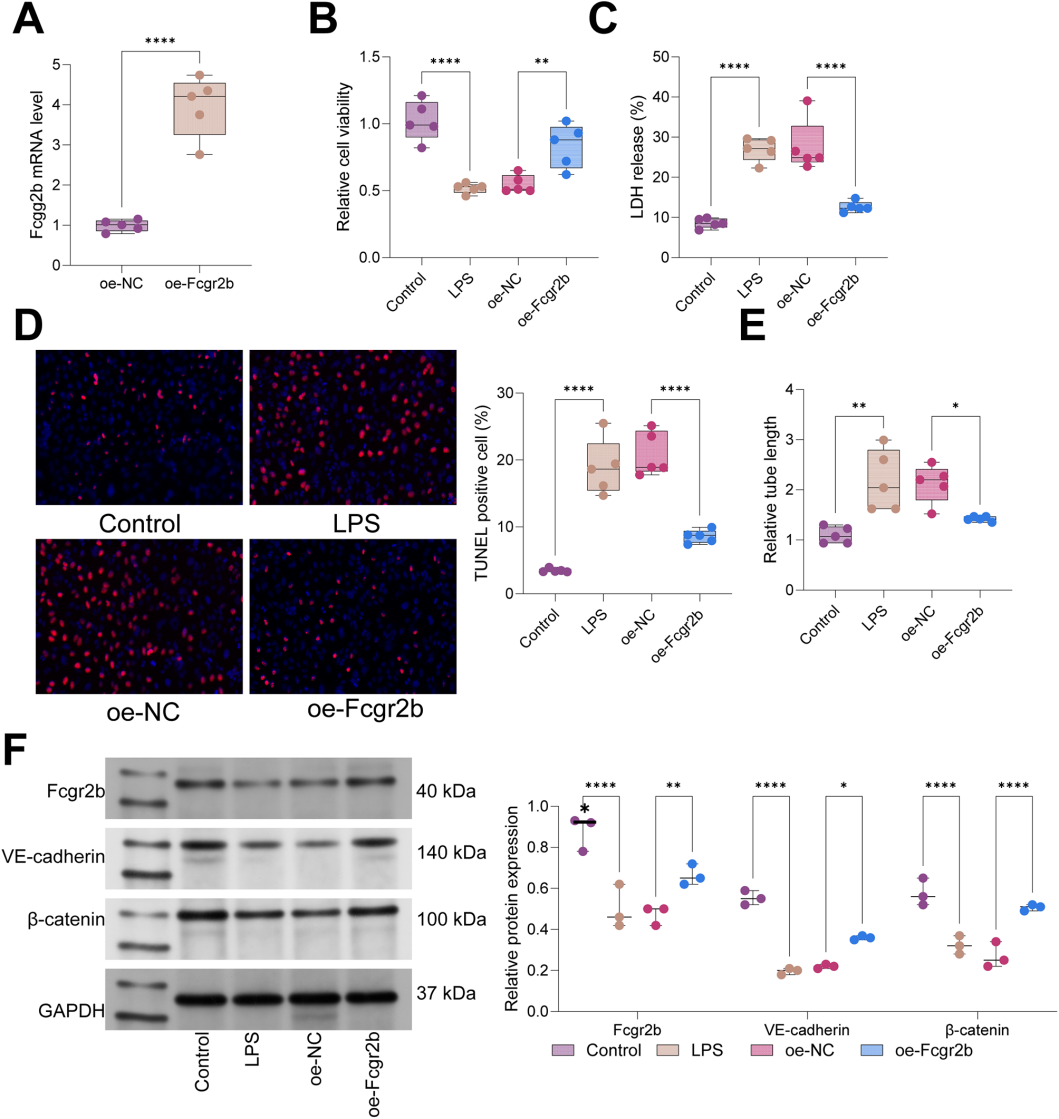
LPS-induced cell injury in PMVECs was restrained by overexpressing Fcgr2b. *Notes* **A**, RT-qPCR for Fcgr2b level in PMVECs; **B**, CCK-8 assay for the activity of PMVECs in each group; **C**, detection of LDH release in each group of PMVECs; **D**, TUNEL assay for the apoptosis of PMVECs in each group; **E**, tubule formation assay for the angiogenic capacity of PMVECs in each group; **F**, western blot for Fcgr2b, VE-cadherin, and β-catenin expression in each group of PMVECs. **N = 3 ~ 5**. Differences between two groups were compared using the *t*-test (**A**), and differences among multiple groups were compared using one-way (**B**-**E**) or two-way (**F**) analysis of variance with Tukey’s post-hoc test. LPS, lipopolysaccharide; PMVECs, pulmonary microvascular endothelial cells; LDH, lactate dehydrogenase

The effects of Fcgr2b on LPS-induced cellular injury in PMVECs were further investigated. PMVECs were transduced with a lentivirus encoding overexpression (oe) Fcgr2b, and the efficiency of transduction was confirmed via RT-qPCR (Fig. 4A). Following transduction, PMVECs were treated with LPS. CCK-8 assays demonstrated that LPS reduced PMVEC viability, an effect that was reversed by oe-Fcgr2b transduction (Fig. 4B). LPS treatment increased lactate dehydrogenase (LDH) release, indicative of heightened cytotoxicity, which was mitigated by oe-Fcgr2b transduction (Fig. 4C). TUNEL assays revealed that LPS treatment elevated apoptosis rates in PMVECs, whereas oe-Fcgr2b transduction counteracted the pro-apoptotic effect of LPS on PMVECs (Fig. 4D). Moreover, LPS treatment enhanced the angiogenic capacity of PMVECs, a response that was attenuated by oe-Fcgr2b (Fig. 4E). Western blot analysis showed that LPS treatment decreased the expression of Fcgr2b and the intercellular junction-associated proteins VE-cadherin and β-catenin, while oe-Fcgr2b transduction increased the expression of Fcgr2b, VE-cadherin, and β-catenin in LPS-treated PMVECs (Fig. 4F). In summary, Fcgr2b overexpression mitigated LPS-induced cellular injury in PMVECs.

### Elk1 was highly expressed and impeded Fcgr2b transcription in ARDS models

**Fig. 5:**
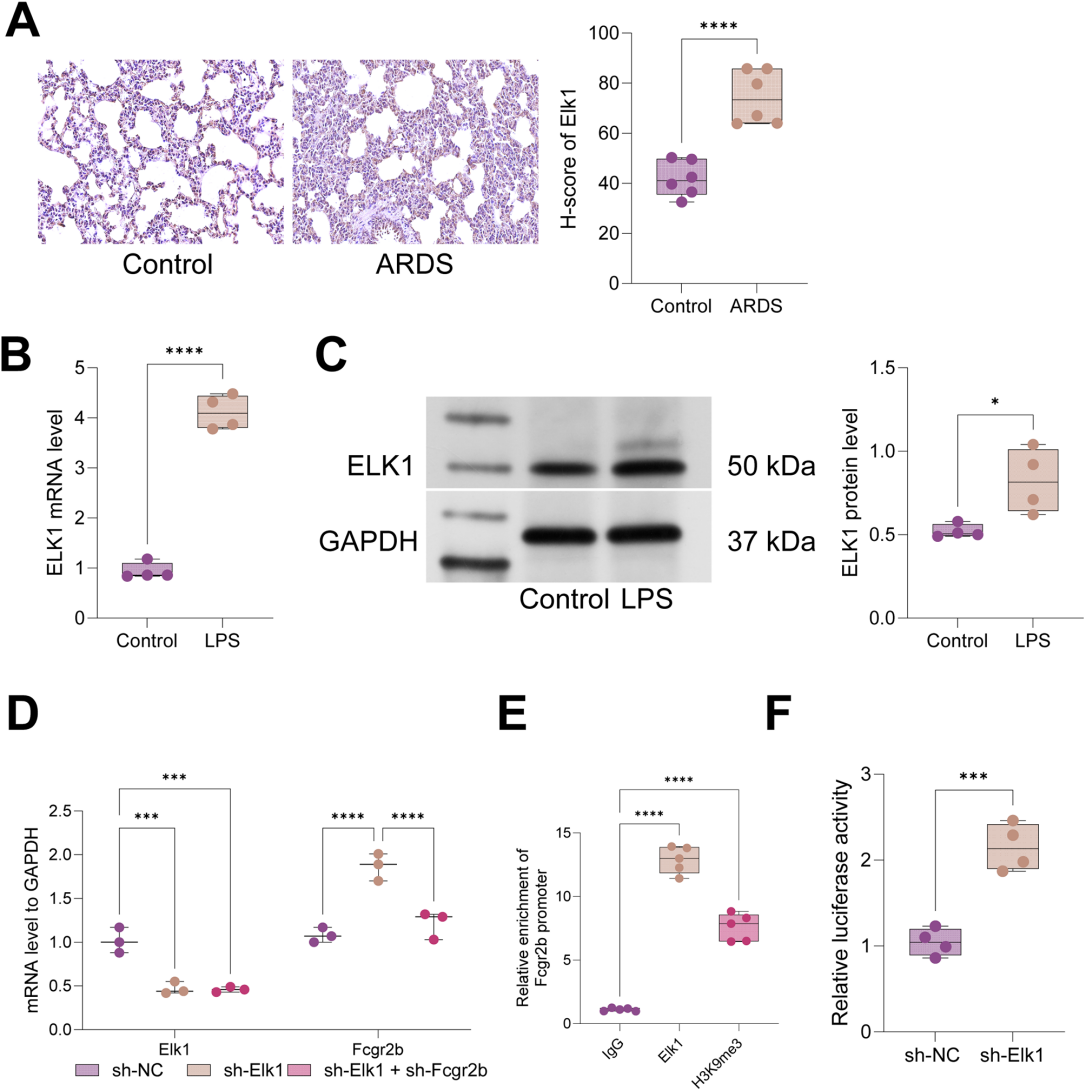
ARDS models have high Elk1 expression, which represses Fcgr2b transcription. *Notes* **A**, Immunohistochemistry to examine Elk1 expression in lung tissues of ARDS rats (*n* = 6/group); **B**-**C**, RT-qPCR and western blot to determine Elk1 expression in LPS-induced PMVECs (*N* = 3); **D**, RT-qPCR to detect Elk1 and Fcgr2b expression in PMVECs after lentiviral infection and LPS induction (*N* = 3); **E**, ChIP-qPCR to assess the binding relationship between Elk1/H3K9me3 and Fcgr2b promoter (*N* = 3); **F**, dual-luciferase reporter assay to evaluate the transcriptional regulation of Elk1 to Fcgr2b (*N* = 3). Differences between two groups were compared using the *t*-test (**A**-**C**, **F**), and differences among multiple groups were compared using one-way (**E**) or two-way (**D**) analysis of variance with Tukey’s post-hoc test. ARDS, acute respiratory distress syndrome; LPS, lipopolysaccharide; PMVECs, pulmonary microvascular endothelial cells; H3K9me3, histone 3 lysine 9 trimethylation

Elk1, a transcription factor potentially regulating Fcgr2b, was identified through ChIP-seq analysis. Immunohistochemical assays revealed elevated Elk1 expression in the lung tissues of LPS-induced ARDS rats (Fig. 5A). Similarly, LPS-induced PMVECs exhibited increased Elk1 expression (Fig. 5B-C). Subsequently, we investigated the regulatory relationship between Elk1 and Fcgr2b in ARDS. PMVECs were transduced with relevant lentiviruses. RT-qPCR analyses showed that sh-Elk1 transduction reduced Elk1 expression and increased Fcgr2b expression, whereas further sh-Fcgr2b transduction decreased Fcgr2b expression without affecting Elk1 expression in PMVECs (Fig. 5D). ChIP-qPCR results demonstrated significant enrichment of Fcgr2b promoters on Elk1 and H3K9me3 compared to IgG (Fig. 5E). Dual-luciferase reporter assays indicated that sh-Elk1 transduction enhanced the luciferase activity of the Fcgr2b promoter (Fig. 5F). In conclusion, Elk1 was highly expressed in ARDS models and suppressed Fcgr2b transcription through the recruitment of H3K9me3.

### Knockdown of Elk1 alleviated the infiltration of Th17 cells and lung tissue injury in ARDS rats by upregulating Fcgr2b.

**Fig. 6:**
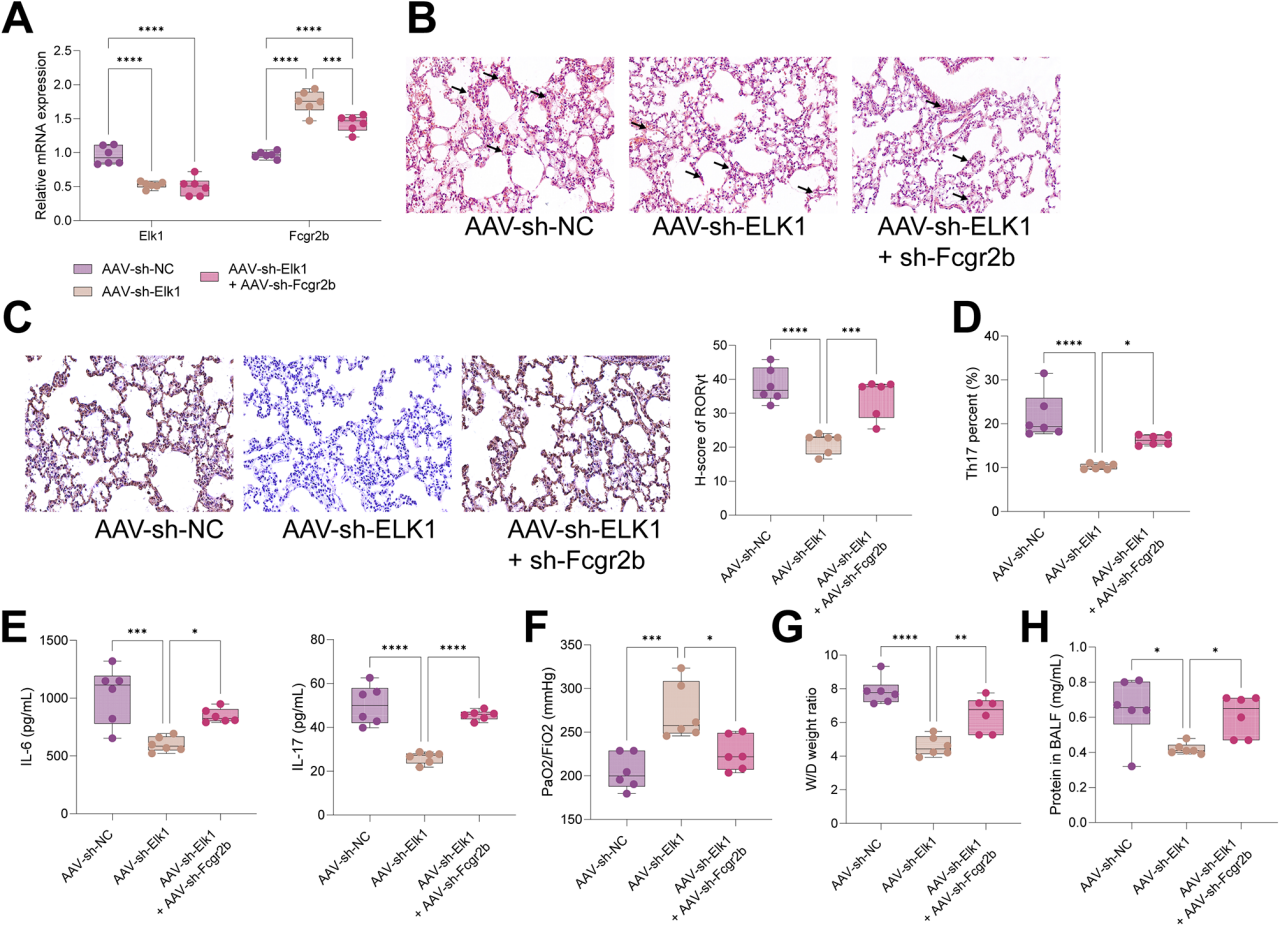
Downregulation of Elk1 increases Fcgr2b level to alleviate the infiltration of Th17 cells and lung tissue injury in ARDS rats. *Notes* **A**, RT-qPCR to examine Elk1 and Fcgr2b expression in lung tissues of ARDS rats; **B**, HE staining to evaluate the pathomorphology of lung tissues of ARDS rats; **C**, immunohistochemistry to measure RORγt expression in lung tissues of ARDS rats; **D**, flow cytometry to detect the proportion of Th17 cells in BALF of ARDS rats; **E**, ELISA to determine IL-17 and IL-6 expression in BALF of ARDS rats; **F**, the PaO2/FiO2 ratio of ARDS rats; **G**, the lung W/D weight ratio of ARDS rats; **H**, the protein concentration in BALF of ARDS rats (*n* = 6/group). Differences among multiple groups were compared using one-way (**C**-**H**) or two-way (**A**) analysis of variance with Tukey’s post-hoc test. ARDS, acute respiratory distress syndrome; PaO2/FiO2, arterial partial pressure of oxygen/fraction of inspired oxygen; W/D, wet/dry; BALF, bronchoalveolar lavage fluid

To examine the actions of Elk1/Fcgr2b in ARDS, rats were treated with AAV and induced with LPS. RT-qPCR results demonstrated that AAV-sh-Elk1 treatment diminished Elk1 expression and upregulated Fcgr2b expression, and further AAV-sh-Fcgr2b treatment diminished Fcgr2b expression (Fig. 6A). HE staining showed that AAV-sh-Elk1 alleviated injury and reduced inflammatory infiltration in lung tissues of ARDS rats, while further AAV-sh-Fcgr2b treatment aggravated lung tissue injury in ARDS rats (Fig. 6B). In addition, AAV-sh-Elk1 treatment declined RORγt expression in lung tissues of ARDS rats, which was reversed after AAV-sh-Fcgr2b treatment (Fig. 6C). Flow cytometry results illustrated that the proportion of Th17 cells in BALF was reduced by AAV-sh-Elk1 but elevated after further AAV-sh-Fcgr2b treatment (Fig. 6D). Moreover, AAV-sh-Elk1 treatment depressed IL-17 and IL-6 expression in lung tissues of ARDS rats, which was negated by AAV-sh-Fcgr2b treatment (Fig. 6E). AAV-sh-Elk1 treatment upregulated the PaO2/FiO2 ratio and alleviated pulmonary edema, whereas AAV-sh-Elk1 and AAV-sh-Fcgr2b treatments diminished the PaO2/FiO2 ratio and exacerbated pulmonary edema (Fig. 6F-G). Also, AAV-sh-Elk1 declined protein concentration in BALF, while proceeding AAV-sh-Fcgr2b treatment resulted in a rebound in protein concentration (Fig. 6H). Summarily, downregulation of Elk1 alleviated the infiltration of Th17 cells and lung tissue injury in ARDS rats, which was counteracted by downregulating Fcgr2b.

### Knockdown of Elk1 alleviated LPS-induced cell injury in PMVECs by upregulating Fcgr2b

**Fig. 7:**
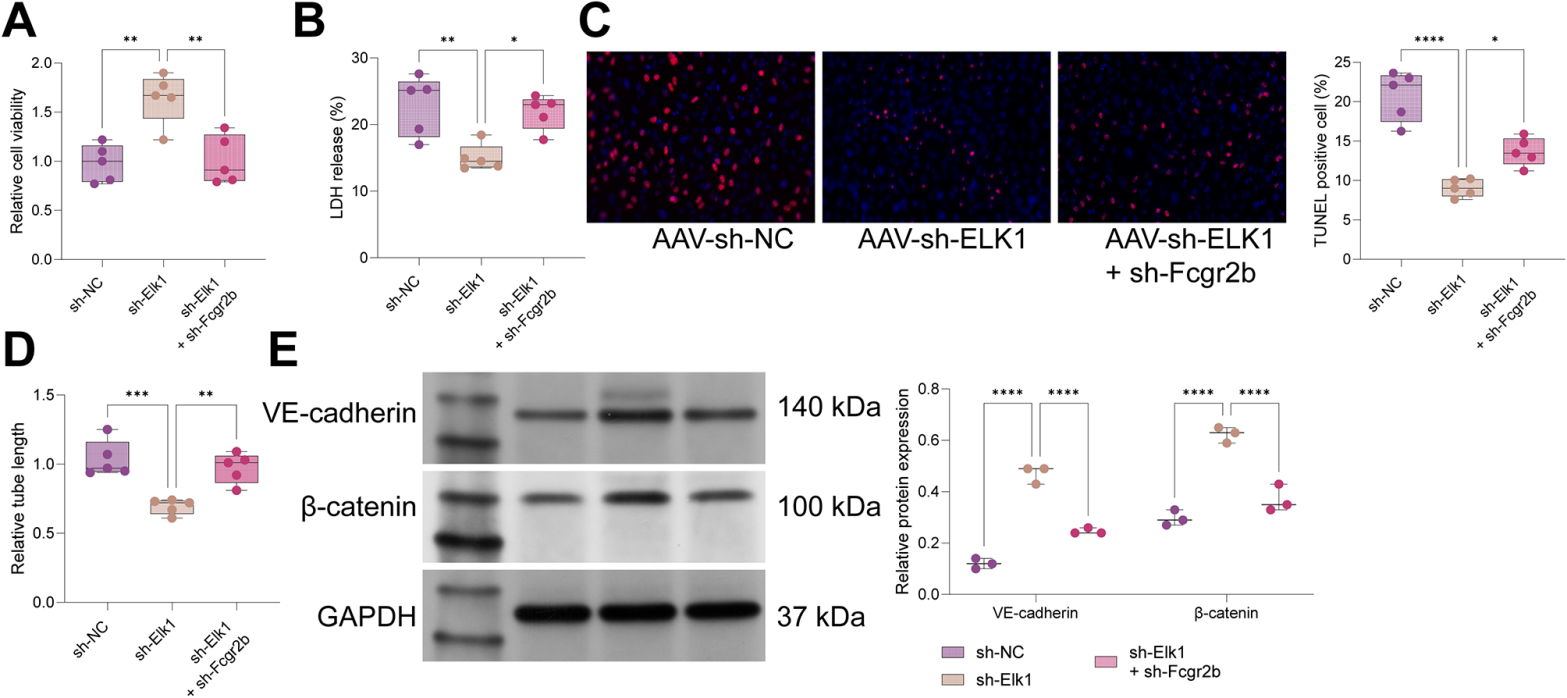
Fcgr2b downregulation represses the protective effect of Elk1 knockdown on LPS-induced cell injury in PMVECs. *Notes* **A**, CCK-8 assay for cell activity of LPS-induced PMVECs; **B**, detection of LDH release in LPS-induced PMVECs; **C**, TUNEL assay for apoptosis of LPS-induced PMVECs; **D**, tubule formation assay for the angiogenic capacity of LPS-induced PMVECs; **E**, western blot for VE-cadherin and β-catenin protein expression in LPS-induced PMVECs. *N* = 3. Differences among multiple groups were compared using one-way (**A**-**D**) or two-way (E) analysis of variance with Tukey’s post-hoc test. LPS, lipopolysaccharide; PMVECs, pulmonary microvascular endothelial cells; LDH, lactate dehydrogenase

Following lentiviral transduction and LPS treatment of PMVECs, the impact of the Elk1/Fcgr2b axis on LPS-induced cellular injury was assessed. The findings indicated that sh-Elk1 transduction improved the viability of LPS-treated PMVECs, an effect that was negated by subsequent sh-Fcgr2b transduction (Fig. 7A). sh-Elk1 transduction reduced lactate dehydrogenase (LDH) release and mitigated cytotoxicity in LPS-treated PMVECs, effects that were reversed by sh-Fcgr2b transduction (Fig. 7B). Furthermore, sh-Elk1 transduction decreased apoptosis and the angiogenic capacity of LPS-treated PMVECs, with these effects being nullified by additional sh-Fcgr2b transduction (Fig. 7C-D). Additionally, sh-Elk1 transduction increased the expression of VE-cadherin and β-catenin proteins, which was diminished by further sh-Fcgr2b transduction (Fig. 7E). In summary, Elk1 knockdown ameliorated LPS-induced cellular injury in PMVECs by upregulating Fcgr2b.

## Discussion

Infection with gram-negative bacteria is a primary cause of ALI. LPS, a key component of the outer membrane of these bacteria, triggers inflammatory responses and lung injury. As a result, LPS has been utilized to induce ALI, establishing it as a recognized model for ARDS research (Li et al. 2021). In this study, we established an ARDS rat model and a cell injury model by administering LPS to rats and PMVECs. It is well understood that ALI and ARDS are characterized by severe dysregulated inflammation, increased pulmonary vascular permeability, decreased lung compliance, interstitial pulmonary and alveolar edema, refractory hypoxemia, and hypoxic respiratory failure (D’Alessio 2018; Liu et al. 2022a, b). The pathophysiological mechanisms underlying ALI/ARDS involve the hyperaggregation and activation of platelets and leukocytes, enhanced permeability of the alveolar-capillary endothelial and epithelial barriers, infiltration by inflammatory cells, and the accumulation of protein-rich edematous fluid in the extravascular space (Liu et al. 2022a, b; He et al. 2021). Th17 cells, a subset of CD4 + T cells, secrete IL-17 and play a pivotal role in mediating lung inflammation, contributing to the development of ARDS (Cheng et al. 2022; Xue et al. 2022). In this study, lung specimens from ARDS models underwent comprehensive pathomorphological analysis, focusing on edema, glycogen deposition, fibrosis, and inflammatory cell infiltration. Additionally, BALF analyses were performed to examine Th17 cell infiltration, inflammatory responses, and changes in microvascular permeability as markers of lung damage. Moreover, the activity, cytotoxicity, apoptotic behavior, and angiogenic capacity of LPS-stimulated PMVECs were quantitatively evaluated to assess cellular injury. Our results reveal that the Elk1/Fcgr2b signaling axis significantly contributes to increased Th17 cell infiltration, enhanced inflammatory responses, and aggravated lung injury in LPS-induced ARDS models.

Fcgr2b, the only inhibitory FcR, governs multiple aspects of inflammatory and immune responses (Verbeek et al. 2019). As stated, Fcgr-/- mice show resistance to transfusion-associated ALI induced by MHC class I mAb (Looney et al. 2006), implying that Fcgr receptors exert effects in ALI. Interestingly, the interaction between Fcgr2a and the IL-8-formed anti-IL-8 autoantibody: IL-8 complexes affects neutrophil apoptosis to result in the development of ALI (Allen and Kurdowska 2014). Fcgr2b-/- mice are hyperreactive to several pathogen molecules like LPS and pneumococcal antigens (Saisorn et al. 2021). Yet, the role of Fcgr2b in ALI/ARDS is still unclear. Significantly, a deficiency in Fcgr2b intensifies fibrosis, inflammation, and lung dysfunction in mice subjected to silica exposure, suggesting that Fcgr2b serves as a protective factor against progressive fibrosing interstitial lung disease (Zhang et al. 2023). In this study, reduced expression of Fcgr2b was detected in the lung tissues of ARDS rats and LPS-stimulated PMVECs. Overexpression of Fcgr2b mitigated ALI in ARDS rats by decreasing inflammatory cell infiltration, expression of inflammatory factors, microvascular permeability, and edema in lung tissues. Additionally, Fcgr2b overexpression attenuated LPS-induced cellular injury in PMVECs by improving cell activity and angiogenic potential while reducing apoptosis and cytotoxicity. Subsequent ChIP-seq analysis identified Elk1 as a potential regulator of Fcgr2b, with our experiments demonstrating that Elk1 inhibited Fcgr2b transcription through the recruitment of H3K9me3.

Elk1, an X-linked transcription factor, is ubiquitously expressed across various organs and cell types, regulating a wide array of biological processes (Cairns et al. 2020). Numerous studies have revealed that Elk1 is implicated in various lung disorders, playing roles such as mediating the sexually dimorphic proliferative response of pulmonary arterial endothelial cells in pulmonary arterial hypertension, promoting the growth of lung cancer cells, and contributing to the progression of idiopathic pulmonary fibrosis (Predescu et al. 2022; Li et al. 2022; Tatler et al. 2016). Arsenite exposure leads to a decrease in occludin levels in BEAS-2B cells and rat lungs via the reactive oxygen species (ROS)/p38 Mitogen-activated protein kinase (MAPK) and ROS/extracellular signal-regulated kinase (ERK)/Elk1/myosin light chain kinase (MLCK) signaling pathways, consequently reducing lung permeability (Liu et al. 2020). Of note, Elk1 is a component of the MAPK pathway which is engaged in the attenuation of LPS-induced ALI in mice by dexmedetomidine (Xu et al. 2015). Elk1 levels are elevated in ventilator-induced lung injury, and the absence of Elk1 reduces edema, necrosis, atelectasis, hyaline membrane formation, hemorrhage, inflammation, and microvascular permeability, thereby mitigating lung injury associated with mechanical ventilation (Tao et al. 2021). Similarly, this study revealed an increase in Elk1 expression in LPS-induced ALI/ARDS models. Moreover, the downregulation of Elk1 ameliorated LPS-induced ALI/ARDS in rats and PMVECs, an effect that was negated by the downregulation of Fcgr2b.

While this study provides valuable insights into the role of the Elk1/Fcgr2b axis in the pathogenesis of ALI/ARDS and highlights the potential therapeutic implications of targeting this pathway, several limitations should be considered. Firstly, our research primarily focused on LPS-induced ALI/ARDS models, which represent only one aspect of the complex pathophysiology of these conditions. Future studies exploring the Elk1/Fcgr2b axis in other models of ALI/ARDS, such as those induced by different pathogens or injury mechanisms, would further validate the generalizability of our findings. Secondly, although we demonstrated a mechanistic link between Elk1 and Fcgr2b expression, additional experiments elucidating the precise signaling pathways involved in this regulation would provide deeper insights into the molecular mechanisms underlying our observations. Moreover, investigating the expression and function of Elk1 and Fcgr2b in clinical samples from ALI/ARDS patients could validate the relevance of our findings in human disease.Lastly, our research primarily evaluated the effects of Elk1/Fcgr2b modulation on the acute phase of ALI/ARDS development. Long-term studies assessing the sustained effects of targeting this pathway on disease progression, resolution, and potential adverse effects are warranted. In summary, while our findings contribute to the understanding of ALI/ARDS pathophysiology and offer promising therapeutic targets, further investigations addressing the aforementioned limitations are necessary to advance the translational potential of our research.

In conclusion, this research for the first time identified reduced Fcgr2b expression in ALI/ARDS models. Furthermore, we discovered that Elk1 suppresses Fcgr2b transcription through the recruitment of H3K9me3, and that overexpression of Fcgr2b mitigates ALI/ARDS by reducing edema, the inflammatory response, and microvascular permeability. Ultimately, we conclude that the Elk1/Fcgr2b axis exacerbates LPS-induced ALI/ARDS, offering insights that may support the development of effective therapeutic strategies for ALI/ARDS.

## Data Availability

The original contributions presented in the study are included in the article, further inquiries can be directed to the corresponding authors.
